# Secreted factors of *Staphylococcus aureus* promote co-invasion with *Candida albicans* by inducing hypha formation and invasion

**DOI:** 10.1128/aem.01961-25

**Published:** 2026-03-31

**Authors:** Raymond Pasman, Bastiaan P. Krom, Jianbo Zhang, Stanley Brul, Sebastian A. J. Zaat

**Affiliations:** 1 Department of Molecular Biology and Microbial Food Safety, Swammerdam Institute for Life Sciences, University of Amsterdam 215691, Amsterdam, the Netherlands; 2 Department of Preventive Dentistry, Academic Centre for Dentistry Amsterdam, University of Amsterdam and Vrije Universiteit Amsterdam, Amsterdam, the Netherlands; 3 Amsterdam Gastroenterology, Endocrinology and Metabolism, Amsterdam UMC, Location Academic Medical Center, Tytgat Institute for Liver and Intestinal Research 534746 , Amsterdam, the Netherlands; 4 Department of Medical Microbiology and Infection Prevention, Amsterdam Institute for Immunology and Infectious Diseases, Amsterdam UMC, University of Amsterdam 1234, Amsterdam, the Netherlands; Indiana University Bloomington, Bloomington, Indiana, USA

**Keywords:** invasion, *Candida albicans*, *Staphylococcus aureus*, polymicrobial, microbial ecology, virulence factors

## Abstract

Interkingdom interactions between *Candida albicans* and *Staphylococcus aureus* promote lethal dissemination of the bacterium. During this process, *C. albicans* hypha invasion aids *S. aureus* dissemination through Als1p/Als3p-facilitated co-invasion. The effects of *S. aureus* on *C. albicans* hypha formation and invasion are, however, unknown. In this study, we used both liquid mDMEM-DMP as well as a previously constructed semi-solid adaptation of the medium (mDMEM-DMPA) to study the effects of *C. albicans/S. aureus* co-culturing on hypha formation and invasion. Semi-solid-based co-culturing significantly increased colony size and generally increased hypha invasion. Liquid growth-based time-lapse microscopy showed that *S. aureus* significantly promoted both *C. albicans* hypha length and elongation rate. Further semi-solid-based growth results revealed that >3 kDa-secreted *S. aureus* factors were accountable for the increase in *C. albicans* hypha growth. A newly constructed *in vitro* assay confirmed the co-invasion of *S. aureus* during co-culturing and showed that deletion of *C. albicans* Als1p/Als3p abolished the co-invasion of *S. aureus* during co-culturing. In conclusion, our study shows that *S. aureus* affects *C. albicans* virulence by actively stimulating *C. albicans* hypha extension through the production of, presently unknown, secreted factors and sequentially using hypha proteins Als1p and Als3p to co-invade. Therefore, *S. aureus* can stimulate *C. albicans* epithelial invasion even prior to attaching to its hyphae, providing the foundation for subsequent co-invasion.

**IMPORTANCE:**

Epithelial barriers normally protect against invasion and systemic infection by *S. aureus*, but frequently, such infections occur without a port of entry. One route of *S. aureus* epithelial traversal is through co-invasion with the highly invasive *Candida albicans*. Understanding this interaction in detail is of high importance in view of the prevention of these infections. Our study shows how the *S. aureus* and C. *albicans* interaction results in mutual benefit. *S. aureus* appeared to affect *C. albicans* virulence by actively stimulating *C. albicans* hypha extension through the production of, presently unknown, secreted factors and sequentially using hypha proteins Als1p and Als3p to bind to the hyphae and co-invade. These insights are important from a microbial ecological perspective and offer important potential targets for interfering with the interaction and reducing the virulence of these opportunistic pathogens.

## INTRODUCTION

Epithelial barriers protect against invasion and systemic infection of opportunistic pathogens. When this barrier is broken, for example, during surgery, individuals are at risk of developing bloodstream infections (BSIs). One of the most common BSI instigators is *Staphylococcus aureus* (1). Due to the fact that *S. aureus*, although highly virulent, is considered a non-invasive commensal, it requires assistance to traverse human epithelial barriers. However, *S. aureus* BSIs frequently manifest without a reported port of entry (2). Since epithelial invasion is decisive for BSI formation, it is crucial to elucidate these unknown routes of *S. aureus* epithelial traversal. One possible route of *S. aureus* invasion is through co-invasion with the highly invasive but less virulent *Candida albicans*.

*C. albicans* is a common member of the human oral mycobiome (3, 4). While generally present as a commensal, various immune deficiencies allow *C. albicans* to become virulent (5). The most crucial aspect of *C. albicans* pathogenesis is switching from non-invasive yeast growth to invasive hypha growth, allowing the fungus to invade underlying tissues (6, 7). Invasion of *C. albicans* hyphae mainly occurs through active penetration (AP); a combination of hydrostatic pressure (turgor) build-up, which forces hyphae through epithelial layers, and the secretion of lytic enzymes and peptides that actively break down epithelial layers (8–11). When AP is allowed to sufficiently progress, it instigates *C. albicans* BSIs (candidemia) (12). Approximately one in five candidemia cases is known to coincide with bacterial BSIs (13), suggesting that the fungus promotes co-invasion of previously non-invasive bacteria, such as *S. aureus*, as well. Together, the combined presence of highly invasive but less virulent *C. albicans* and the non-invasive but highly virulent *S. aureus* could result in both a highly invasive and highly virulent *S. aureus* BSI.

Accordingly, numerous *in vivo* murine studies have shown that oral and peritoneal co-infections with *C. albicans* and *S. aureus* are significantly more lethal compared to mono-infections and that this effect is mainly attributable to the co-invasion and dissemination of *S. aureus* (14–23). *C. albicans* crucially contributes to this process by breaking epithelial layers (through AP) and sequentially facilitating *S. aureus* co-invasion via binding to hypha agglutinin-like sequence proteins 1 and 3 (Als1 and Als3) (18, 22–25). Although *C. albicans* invasion is vital to the process, the vast majority of *in vitro* studies have been focused on the impact of *C. albicans* on *S. aureus* virulence (14, 26–30), leaving the effects of *S. aureus* on *C. albicans* virulence, especially AP, understudied.

Previously, we developed an *in vitro* semi-solid growth medium (mDMEM-DMPA) able to support mono and co-culturing of *C. albicans* and *S. aureus* (31). The aim of this study was to investigate the impact of *S. aureus* on *C. albicans* hypha formation and (co-)invasion when grown in mDMEM-DMPA. First, we studied the effect of this *in vitro* semi-solid co-culturing on colony expansion and invasion. Next, we evaluated which *C. albicans* factors contributed to *S. aureus* colony expansion and which *S. aureus* factors contributed to *C. albicans* colony/hypha extension and invasion. Finally, we investigated whether *S. aureus*-induced *C. albicans* hypha formation/invasion promotes *S. aureus* co-invasion into a semi-solid growth surface using a constructed model for co-invasion.

## MATERIALS AND METHODS

### Strains and growth conditions

*C. albicans* and *S. aureus* strains used in this study are listed in Table 1. *C. albicans* strains were maintained from glycerol freezer stocks on Sabouraud Dextrose Agar with chloramphenicol (Sigma, 63567). *S. aureus* strains were maintained on mannitol salt phenol red agar (MSA, Sigma, 89579). Regarding *S. aureus* ATCC 12600^GFP^, tetracycline (5 µg/mL, Sigma, T3258) was supplemented to the growth media. For liquid cultures, single colonies were inoculated in tryptic soy broth (TSB; Brunschwig Chemie, 211825) and grown overnight at 37°C, 200 rpm. Cultures were washed using phosphate-buffered saline (DPBS; 137 mM NaCl, 2.7 mM KCl, 10 mM Na_2_HPO_4_, and 1.8 mM KH_2_HPO_4_) and resuspended in mDMEM-DMP (Dulbecco’s modified Eagle’s medium [Sigma D5030], 2.5 g/L dextrose, 1× sodium pyruvate [Gibco 11360070], 1× Glutamax [Gibco 35050061], and 1× MEM non-essential amino acids [Gibco 11140050]), at a final pH of 7.3 and filter-sterilized (31). Where needed, 100 mM of 4-(2-hydroxyethyl)-1-piperazineethanesulfonic acid (HEPES, Gibco 15630056) was added to the medium. Cultures were diluted in mDMEM-DMP to ~2∙10^6^ CFU/mL *C. albicans* and ~2∙10^7^ CFU/mL *S. aureus*. Monocultures were diluted 1:1 with mDMEM-DMP, and co-cultures were obtained by mixing monocultures in a 1:1 ratio.

**TABLE 1:** Strains used in this study

Strain	Description	Reference
*S. aureus* strains		
ATCC 12600	NCTC 8532 wild type	(32)
ATCC 12600^GFP^	ATCC 12600 pMV158 GFP	(33)
Newman	ATCC 25904; exhibits high-level clumping factor production; σ^B+^	(34)
NCTC8325-4	Derivative of NCTC8325, cured of all prophages; carries 11-bp deletion in rsbU	(35)
NCTC8325-4 *agr*	NCTC8325-4 derivative; *agr*::tet, Agr deletion mutant	(36)
*C. albicans* strains		
SC5314	Wild type, clinical isolate	(37)
SC5314 als1/als3 ΔΔ/ΔΔ	als1/als3 ΔΔ/ΔΔ *S. aureus* binding deficient mutant *als1-1Δ*::*FRT/als1-2Δ*::*FRTals3-1Δ*::FRT/*als3-2Δ*::*FRT*	(17)
SC5314 cph1/efg1 ΔΔ/ΔΔ	Cph1/Efg1 hypha-deficient mutant HLC54 *efg1Δ/Δ cph1Δ/Δ ura3Δ::imm434/ura3Δ::imm434 cph1::hisG/cph1::hisG efg1::hisG/efg1::hisG-URA3-hisG*	(38)

### Colony growth assessment

Colony growth was studied on top of mDMEM-DMP agar (0.3% wt/vol) (mDMEM-DMPA) (31). mDMEM-DMPA was prepared by mixing 2× concentrated mDMEM-DMP (either buffered or unbuffered) with sterile 0.6% wt/vol agar (Sphaero Q B.V, A02076) (55°C) in a 1:1 ratio. Directly after mixing, 3 mL of mDMEM-DMPA was added to wells of a six-well plate (Thermo Scientific 140685) and dried under laminar flow. In the case of *C. albicans* monocultures, colony growth was also tested in cultures supplemented with heat-inactivated (HI) *S. aureus* and *S. aureus*-secreted factors. HI *S. aureus* was obtained by first growing an overnight *S. aureus* ATCC 12600 culture in TSB (37°C, 200 rpm), subsequently incubating the culture at 80°C for 20 min, and washing it three times using DPBS. Efficacy of heat inactivation was confirmed by the lack of *S. aureus* growth on TSB plates. HI *S. aureus* was mixed with the *C. albicans* monoculture at a final OD_600nm_ of 0.1. *S. aureus*-secreted factors were acquired by adding 3 mL of *S. aureus* (1∙10^7^ CFU/mL) to wells of a six-well plate, incubating it stationary for 2 h at 37°C to allow adherence of *S. aureus*, washing the wells with DPBS to remove unbound bacteria, adding 3 mL of fresh buffered mDMEM-DMP (100 mM HEPES), and incubating the plates stationary for 72 h at 37°C in a humidified environment. Spent medium of six wells was then collected and combined and sterilized using a 0.2-µm filter (Sarstedt, 83.1826.001). Using 3 kDa centrifugal filter units (Pall, MAP003C37), the collected medium was split into a fraction containing molecules larger than 3 kDa and a fraction containing all remaining molecules smaller than 3 kDa. Both fractions were supplemented to the original volume with mDMEM-DMP and diluted in *C. albicans* cultures (1∙10^7^ CFU/mL) in a 1:9 ratio before culturing. For all conditions, a 2 µL drop of culture was carefully pipetted in the center of an mDMEM-DMPA filled well and dried under laminar flow. Finally, plates were incubated stationary at 37°C for 72 h (in a humidified environment) and imaged using a FastGene FAS-V Geldoc system. Colony areas (mm^2^) were measured using ImageJ (version 1.53q), tested for normality using a Shapiro-Wilk test, and differences in sizes were tested for significance using either a one-way ANOVA or Kruskal-Wallis test (depending on data normality), combined with a Tukey post hoc analysis using GraphPad Prism (8.3.0). Data were visualized as boxplots using Microsoft Excel for Microsoft 365 MSO (Version 2303 Build 16.0.16227.20202) 64-bit. All conditions were tested in at least three biological as well as technical replicates.

To further test the effects of secreted factors, 100 µL of cultures were inoculated at a centroid distance of approximately 2.5 cm in order to grow without overlap, dried under laminar flow, and imaged as described above.

### Colony invasion assessment

The invading proportion of the colony was imaged as previously described, with minor adaptations (39). Monocultures of *Candida* and co-cultures containing *S. aureus* ATCC 12600^GFP^ were grown as described above. To wash off the non-adherent section of the colonies, each colony was exposed to a consistent flow of 1 mL sterile MQ water five times, after which colonies were re-imaged, all using a Nikon SMZ1000 coupled with a Zeiss AxioCam MRc, Nikon LH-M100C-1, and a 495nm GFP filter. Images were constructed using ImageJ (version 1.53q). All conditions were tested in at least three biological as well as technical replicates.

### Hypha growth

Hypha growth was assessed by adding 200 µL of DPBS containing 2.5∙10^4^ CFU/mL *C. albicans* SC5314 wildtype, with or without various *S. aureus* ATCC 12600^GFP^ densities, to wells of an Ibidi treated polymer coverslip µ-Plate 96 Well Black plates (Ibidi). Plates were incubated stationary at 37°C for 2 h, washed with PBS, and resuspended in fresh mDMEM-DMP. Concerning co-cultures, various *S. aureus* ATCC 12600^GFP^ ratios were added to *C. albicans* monocultures: 2.5∙10^4^ CFU/mL (1:1), 2.5∙10^5^ CFU/mL (1:10), or 2.5∙10^6^ CFU/mL (1:100) (final concentrations). Plates were incubated for 15 h at 37°C under atmospheric CO_2_ and imaged every 10 min using a Nikon Eclipse Ti equipped with a Nikon Ti Ph3 phase contrast condenser. Connected to it were a Nikon Plan Apo λ Oil Ph3 DM lens (100×, NA = 1.49, T = 23°C), a NIDAQ Lumencor shutter, a Ti XY-and Z drive, and a Hamamatsu C11440-22C camera. All hardware was connected to a computer running NIS-Elements AR 4.50.00 (Build 1117) Patch 03. Hyphal length (µm) progression over time and maximum hyphal length (µm) were measured for 200 hyphae divided over four biological repeats using the segmented line tool inside ImageJ 1.53t. Average hyphal growth rates (µm/h) were calculated per hypha for the first and second 7.5 h using Microsoft Excel. Data were tested for normality using a Shapiro-Wilk test and tested for significance using either a one-way ANOVA or a Kruskal-Wallis test (depending on data normality) combined with a Tukey post hoc analysis using GraphPad Prism (8.3.0). Sequentially, data were visualized using Microsoft Excel for Microsoft 365 MSO (Version 2303 Build 16.0.16227.20202) 64-bit.

### Co-invasion assay

Co-invasion assay plates were prepared by first adding 3 mL of liquid mDMEM-DMPA (55°C) to the wells of a preheated (55°C) six-well plate. Directly after, a layer of mDMEM-DMPA (either 500, 600, 700, 800, 900, or 1,000 µm thick) was added to the inside of a pre-heated (55°C) 8-µm *trans*-well insert (CellQart 9308012). Before both agar layers solidified, inserts were placed into the mDMEM-DMPA-filled wells, merging both agar layers, after which the agar was solidified under laminar flow. Finally, the agar was inoculated with mono- and co-culture *C. albicans* wild type, *C. albicans* als1/als3 ΔΔ/ΔΔ, and *S. aureus* ATCC 12600 and grown as described above. Following growth, the *trans*-well inserts were removed from the wells, after which the bottom site of the inserts was scraped with cell scrapers (Greiner, 391-3010), and the collected scrapings were spread over either *C. albicans*-selective (SDA) or *S. aureus*-selective (MSA) plates. The selective plates were sequentially grown overnight at 37°C and checked for growth. All conditions were tested in at least three biological as well as technical replicates.

## RESULTS

### Co-culturing of *C. albicans* and *S. aureus* significantly increases colony size and agar invasion

**Fig 1:**
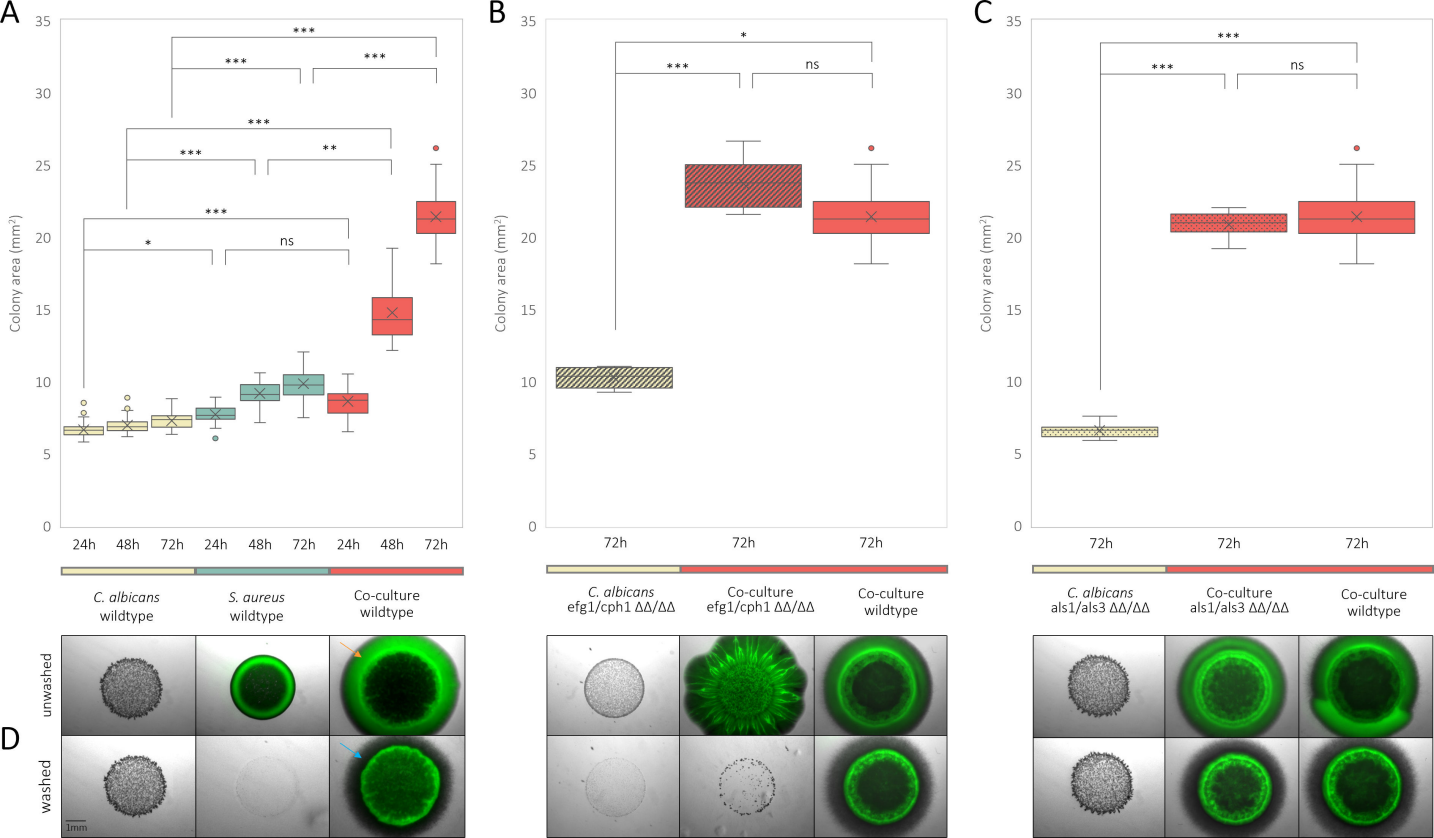
Colony area (mm^2^) progression over the course of 72 h of monocultures (*C. albicans*: yellow, *S. aureus*: blue) and co-cultures (red) (**A**). Colony areas (mm^2^) measured at 72 h of *C. albicans efg1/cph1* monocultures (yellow dashed) and co-cultures containing *S. aureus* ATCC 12600 wild type (red dashed) (**B**). Colony areas (mm^2^) measured at 72 h of *C. albicans* als1/als3 ΔΔ/ΔΔ monocultures (yellow dotted) and co-cultures containing *S. aureus* ATCC 12600 wild-type (red dotted) (**C**). Colony sections present before and after washing (**D**) with each colony corresponding to the condition represented above in A, B, and C, albeit containing *S. aureus* ATCC 12600^GFP^. Because semi-solid growth substratum anchors the fungal cells, provided they produce hyphae, washing does not affect the colonies of the *C. albicans* wild type and its als1/als3 ΔΔ/ΔΔ mutant strain monocultures, leaving their images nearly identical to their “unwashed” condition. The orange arrow corresponds to the GFP signal of non-attached *S. aureus* at the air/agar interface that is washed away. *C. albicans* hyphae, indicated by the blue arrow, are visible underneath this washed-away *S. aureus* section. All conditions were tested in biological and technical triplicate and tested for significance using either a one-way ANOVA or Kruskal-Wallis test (depending on data normality) combined with a Tukey’s post hoc analysis (*P ≤ 0.05, **P ≤ 0.01, ***P ≤ 0.001, ns: not significant).

To investigate the effect of co-culturing on colony growth, mono- and co-cultures of *C. albicans* SC5314 (wild type*,* efg1/cph1 ΔΔ/ΔΔ, and als1/als3 ΔΔ/ΔΔ) and *S. aureus* were grown on semi-solid mDMEM-DMPA (Fig. 1A through C; Fig. S1). Co-culture colonies containing wild-type *C. albicans* and *S. aureus* grew significantly larger after 48 h compared to all wild-type monoculture colonies (*P* < 0.001) (Fig. 1A). To study the contribution of *Candida* hypha formation to this co-culture colony expansion, *S. aureus* was co-cultured with a hypha-deficient *C. albicans* efg1/cph1 ΔΔ/ΔΔ mutant (Fig. 1B). Colony sizes of co-cultures containing *C. albicans* efg1/cph1 ΔΔ/ΔΔ did not differ from co-cultures containing *C. albicans* wild type. To investigate the effect of *S. aureus* binding to *C. albicans* hypha Als1p and Als3p, *S. aureus* was co-cultured with an Als1p/Als3p-deficient *C. albicans* als1/als3 ΔΔ/ΔΔ mutant (Fig. 1C). Again, als1/als3 ΔΔ/ΔΔ co-culture colony sizes did not differ from the wild type co-culture colonies. Together, this indicates that the observed colony size increase is independent of *C. albicans* hypha formation and Als1p/Als3p binding.

To visualize the invading proportion of each condition, colonies were grown using *S. aureus* ATCC 12600^GFP^ and imaged before and after washing (Fig. 1D). Although monoculture wild-type *S. aureus* colonies were readily washed off, wild-type *C. albicans* monocultures and co-culture colonies remained attached (Fig. 1D). However, the *S. aureus* GFP signal on the outer rim of these co-culture colonies was washed away (Fig. 1D, orange arrow), indicating that at the colony edge, *S. aureus* was partly unattached to the hypha colony and/or agar. Underneath the washed-away *S. aureus*, a similarly sized area of *C. albicans* hyphae remained visible (Fig. 1D, blue arrow), showing that *C. albicans* equally contributes to colony expansion, albeit underneath the agar surface. Regarding mono- or co-cultures containing the hypha-deficient *C. albicans* efg1/cph1 ΔΔ/ΔΔ mutant, none of the colonies remained attached to the agar when subjected to the washing procedure. This showed that *C. albicans* hyphae were accountable for the attachment/invasion to/into the agar and, thereby, the intra-agar colony expansion. To investigate whether the attachment of *S. aureus* to the hypha colony was als1/als3 dependent, co-cultures containing *C. albicans* als1/als3 ΔΔ/ΔΔ were grown and tested. After washing *C. albicans* als1/als3 ΔΔ/ΔΔ*–S. aureus* ATCC 12600^GFP^ co-culture colonies, *S. aureus* GFP signal remained present, proving that *S. aureus* integration and attachment to the colony was Als1p and Als3p independent.

Altogether, co-culturing of *C. albicans* and *S. aureus* significantly increased colony expansion both above and within the agar. Although the expansion at the air/agar interface was mainly attributable to *S. aureus*, the intra-agar expansion was mainly attributable to invading *C. albicans* hyphae. Furthermore, *C. albicans* hyphae allowed for *S. aureus* attachment to the colony/agar during washing in an Als1p/Als3p-independent manner, although Als1p/Als3p are essential for binding of *S. aureus* to *C. albicans* hyphae.

### pH stabilization by *C. albicans* promotes *S. aureus* colony expansion in an Agr-independent manner

**Fig 2:**
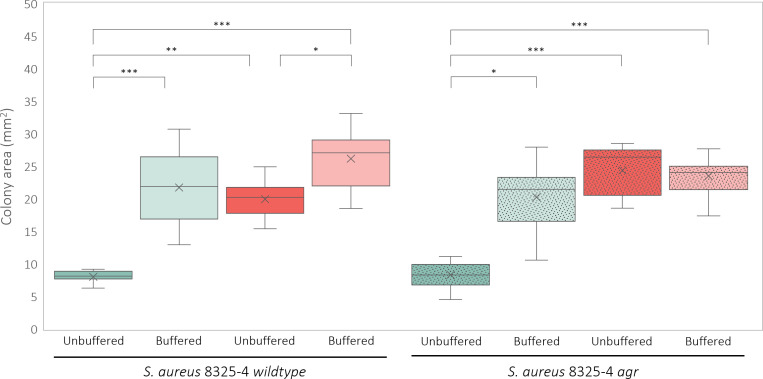
Colony sizes (mm^2^) of *S. aureus* 8325-4 (blue) wild-type (undotted) and *agr* (dotted) and corresponding co-cultures containing *C. albicans* SC5314 (red) after 72 h of unbuffered (dark colored) or buffered (100 mM HEPES, light colored) growth. All conditions were tested in at least biological and technical triplicate and tested for significance using a Kruskal-Wallis test combined with a Dunn’s multiple comparison test. Only significant differences are indicated (*P ≤ 0.05, **P ≤ 0.01, ***P ≤ 0.001).

One possible explanation of the observed growth increase of *S. aureus* during co-culturing with *C. albicans* can be pH stabilization. When grown unbuffered, as described above, *S. aureus* growth can be diminished due to medium acidification (31). *C. albicans* alkalinization during planktonic co-culturing is known to counteract this acidification and promote *S. aureus* growth during planktonic culturing (29, 31). To study whether *C. albicans* pH stabilization is contributing to the observed *S. aureus* colony size increase, both mono- and co-cultures of wild-type *C. albicans* and *S. aureus* were grown on mDMEM-DMPA supplemented with 100 mM HEPES and compared to the previously measured *S. aureus* mono- and co-culture colonies grown on unbuffered mDMEM-DMPA (Fig. 2). *S. aureus* monoculture colonies grown on buffered mDMEM-DMPA grew significantly larger (*P* < 0.001) compared to corresponding monoculture colonies grown on unbuffered mDMEM-DMPA. The buffered *S. aureus* monoculture colony sizes did not differ from unbuffered *S. aureus–C. albicans* co-culture colonies, suggesting that the observed *S. aureus* colony size increase is due to pH stabilization. Nevertheless, co-culture colonies grown on buffered mDMEM-DMPA were significantly larger compared to unbuffered co-culture colonies (*P* < 0.050), indicating that either the co-culture colonies’ pH was not fully maintained or that other factors could still contribute to colony expansion.

Neutral pH, as maintained by *C. albicans* during co-culturing, is known to activate the *S. aureus* Agr quorum-sensing system (29). The Agr system, in turn, promotes passive colony expansion of *S. aureus* (colony spreading) (40) through the secretion of phenol-soluble modulins. Therefore, neutral pH induced Agr activation could explain the observed *S. aureus* colony size increase in response to buffering (Fig. 2). To study whether this pH-sensitive Agr system is involved in the observed colony size increase, Agr-deficient *S. aureus* strain (*agr*) mono- and co-cultures, containing wild-type *C. albicans,* were investigated (Fig. 2). All cultures were grown on either unbuffered or buffered (100 mM HEPES) mDMEM-DMPA. No significant differences in colony size were observed between any of the wild-type and their corresponding *agr* mutant conditions (Fig. 2). Comparable to the wild type, *S. aureus agr* colonies grew significantly larger (*P* < 0.05) on buffered mDMEM-DMPA compared to the corresponding monoculture colonies grown on unbuffered mDMEM-DMPA (Fig. 2). Furthermore, *S. aureus agr* monoculture colonies grown on buffered mDMEM-DMPA did not differ compared to both unbuffered or buffered (*P* > 0.99) co-culture colonies (Fig. 2), indicating that the Agr system is not responsible for the previously observed *S. aureus* colony size increase in response to medium buffering (Fig. 2). Noteworthy, the significant increase observed between unbuffered and buffered wild-type co-culture conditions was not observable between the *agr* co-culture conditions, suggesting that the Agr system has a negative impact on colony size increase during unbuffered co-culturing. Thus, during co-culturing, *C. albicans* mainly promotes *S. aureus* colony expansion at the air/agar interface through pH stabilization but not by promoting passive colony expansion through the pH-sensitive staphylococcal Agr system.

### *S. aureus* promotes *C. albicans* hypha growth in a concentration-dependent manner

**Fig 3:**
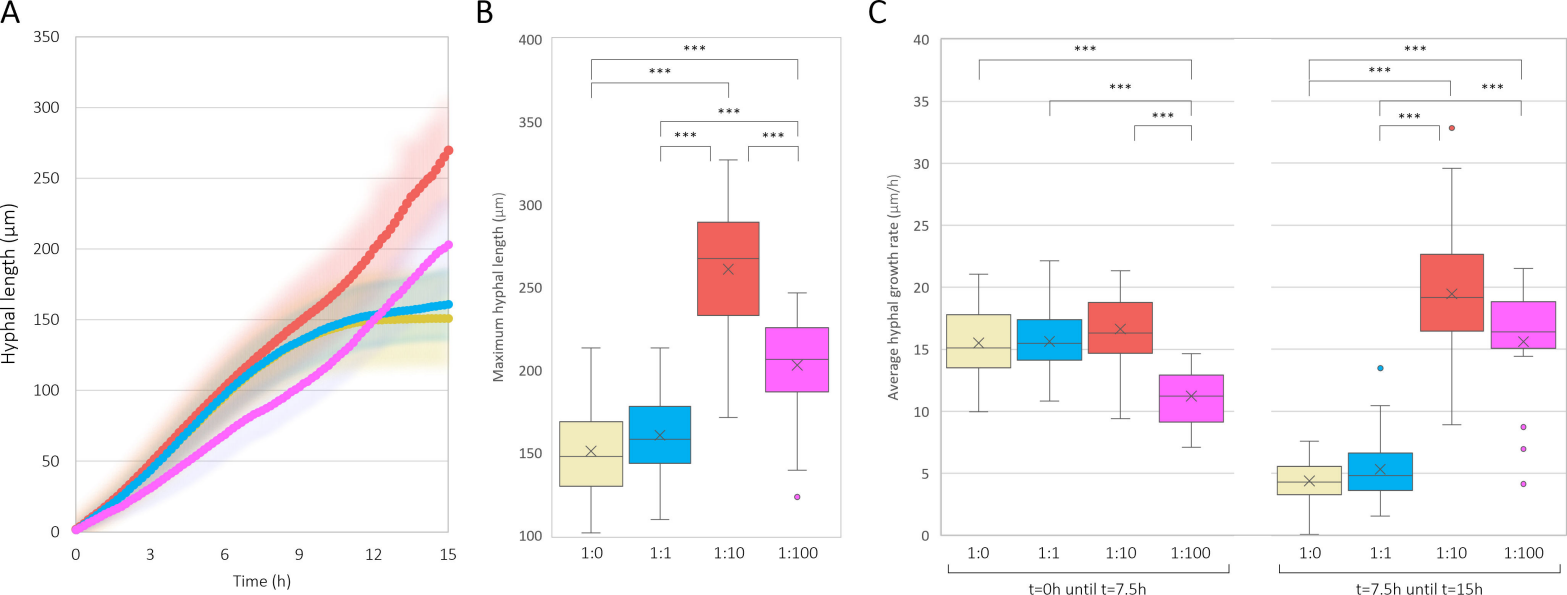
*C*. *albicans* SC5314 wild-type hyphal length (µm) progression of 200 hyphae over the course of 15 h for either monocultures (1:0, yellow) or co-cultures with *S. aureus* in either a 1:1 ratio (blue), 1:10 ratio (red), or 1:100 ratio (pink), standard deviations are represented by correspondingly colored glows (**A**). Derived from this graph, per hypha: maximum hyphal length (µm), (**B**) and average hyphal growth speed (µm/h) (**C**) over the course of the first (C, left) and second (C, right) 7.5 h of growth. Data were tested for significance using either a one-way ANOVA or Kruskal-Wallis test (depending on data normality) combined with a Tukey post-analysis (*P ≤ 0.05, **P ≤ 0.01, ***P ≤ 0.001, ns: not significant). Outliers are visualized as separate points outside of the boxplot.

Aside from the *C. albicans*-stimulated growth of *S. aureus*, *S. aureus* was observed to promote *C. albicans* hypha formation and invasion (Fig. 1D). To investigate the effect of *S. aureus* on *C. albicans* hypha formation, solid surface time-lapse images of unbuffered wild-type *C. albicans* monocultures and co-cultures with various *C. albicans:S. aureus* ATCC 12600^GFP^ ratios were recorded and analyzed first (Fig. 3). Co-culturing of *C. albicans* with *S. aureus* at a ratio of 1:10 and 1:100 resulted in significantly longer hyphae compared to *C. albicans* monocultures and co-cultures with a ratio of 1:1 (*P* < 0.001) (Fig. 3A and B). These observed hypha growth differences are reflected in hypha extension rates (Fig. 3C), which, during 7.5–15 h of growth, nearly halted in monocultures and 1:1 co-cultures but increased in 1:10 and 1:100 co-cultures, proving that *S. aureus* promotes *C. albicans* hypha elongation in a concentration-dependent manner. Nevertheless, co-cultures with a ratio of 1:100 showed significantly shorter *C. albicans* hyphae (*P* < 0.001) compared to 1:10 co-cultures. Moreover, compared to 1:10 co-cultures, extension rates of hyphae during 1:100 co-culturing were significantly reduced during the first 7.5 h (*P* < 0.001) but were no longer different during the last 7.5 h, showing that too high starting concentrations of *S. aureus* likely reduced induction of hypha elongation during the early phase of co-culturing.

Together, these results show that *S. aureus* promotes both the duration of *C. albicans* hypha extension as well as the extension rate on a solid surface in a ratio-dependent manner with an optimal *C. albicans:S. aureus* ratio of 1:10.

### *C. albicans* hypha extension and invasion are stimulated by *S. aureus*-secreted factors

**Fig 4:**
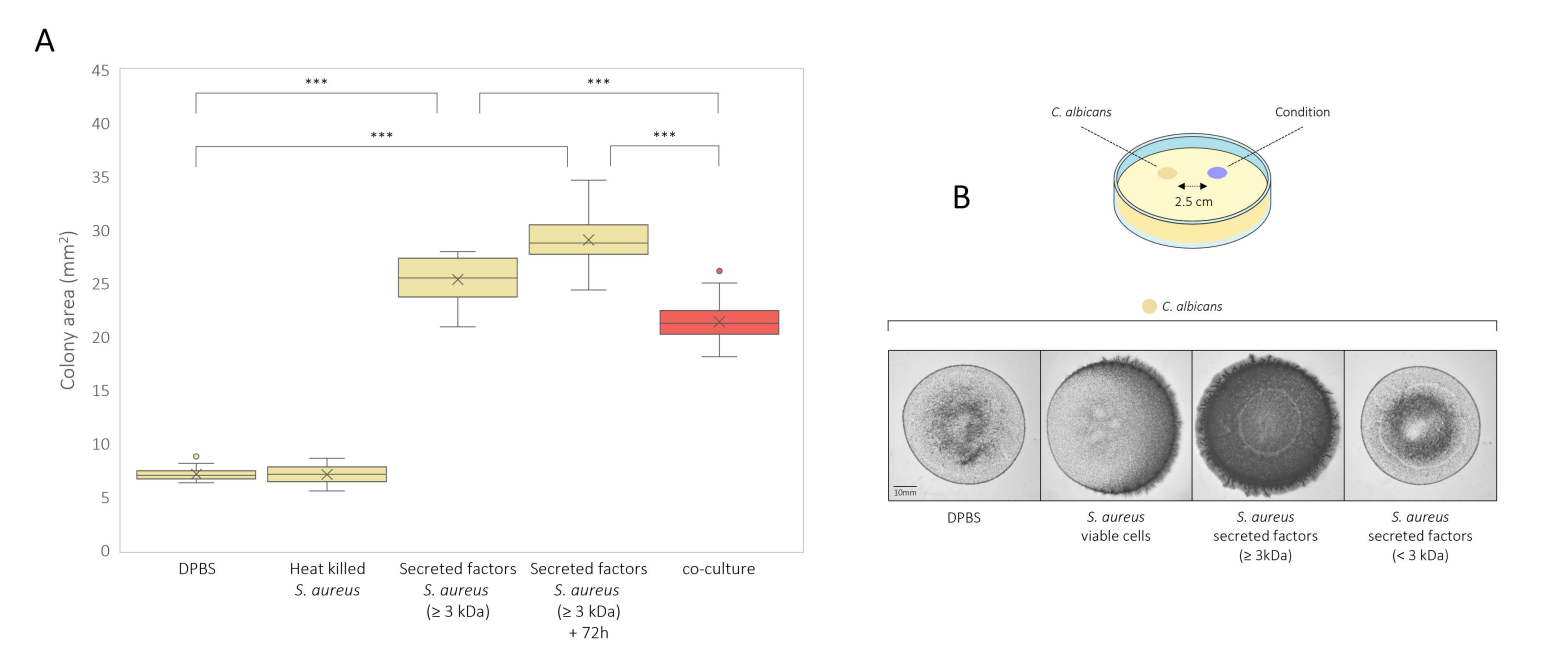
Colony areas (mm^2^) after 72 h of growth of *C. albicans* SC5314 monocultures (yellow) exposed to either heat-killed *S. aureus* ATCC 12600 wild-type (yellow) or *S. aureus* ATCC12600 wild-type-secreted factors with a Mw larger than 3 kDa (yellow), as well as co-cultures (red) containing viable *S. aureus* ATCC12600 wild type (**A**). Wild-type *C. albicans* SC5314 monoculture colonies (72 h) grown separately at a distance of 2.5 cm from DPBS, viable *S. aureus* ATCC12600 wild type, *S. aureus* ATCC12600 wild-type-secreted factors with a Mw larger than 3 kDa or with a Mw smaller than 3 kDa (**B**). All conditions were tested in biological and technical triplicate and tested for significance using either a one-way ANOVA or Kruskal-Wallis test (depending on data normality), combined with a Tukey post hoc analysis (*P ≤ 0.05, **P ≤ 0.01, ***P ≤ 0.001, ns: not significant).

To investigate whether *S. aureus* cells or secreted factors were accountable for the observed hypha growth promotion, *C. albicans* monoculture colonies were grown (on mDMEM-DMPA) together with heat-killed *S. aureus* or secreted *S. aureus* factors (≥ 3 kDa) (Fig. 4A). Addition of DPBS as a control or heat-killed *S. aureus* cells did not impact colony areas of *C. albicans* monocultures. However, supplementation with *S. aureus* secreted factors with a molecular weight (Mw) of ≥3 kDa significantly increased colony area sizes (*P* < 0.001), exceeding those observed during co-culturing. This effect was confirmed by growing *C. albicans* and *S. aureus* monoculture colonies with a 2.5 cm spacing, avoiding direct contact (Fig. 4B). Here, the monoculture colony containing viable *S. aureus* cells stimulated hypha formation in the monoculture colony containing *C. albicans* (Fig. 4B). The observed hypha stimulation was stronger at the *C. albicans* colony side directed toward the staphylococcal colony, indicating that diffused factors from the *S. aureus* colony account for the hypha induction in the *C. albicans* colony (Fig. 4B). Addition of *S. aureus* secreted factors (≥ 3 kDa), at a similar distance, induced hypha formation of the entire *Candida* colony. Addition of DPBS, or *S. aureus*-secreted factors with an Mw of less than 3 kDa, showed no impact on hypha formation.

Thus, the previously observed increase in *C. albicans* hypha extension during co-culturing with *S. aureus* (Fig. 1D) was induced by secreted *S. aureus* factors with a molecular weight greater than 3 kDa.

### *C. albicans* supports *S. aureus* co-invasion in an Als1p/Als3p-dependent manner

**Fig 5:**
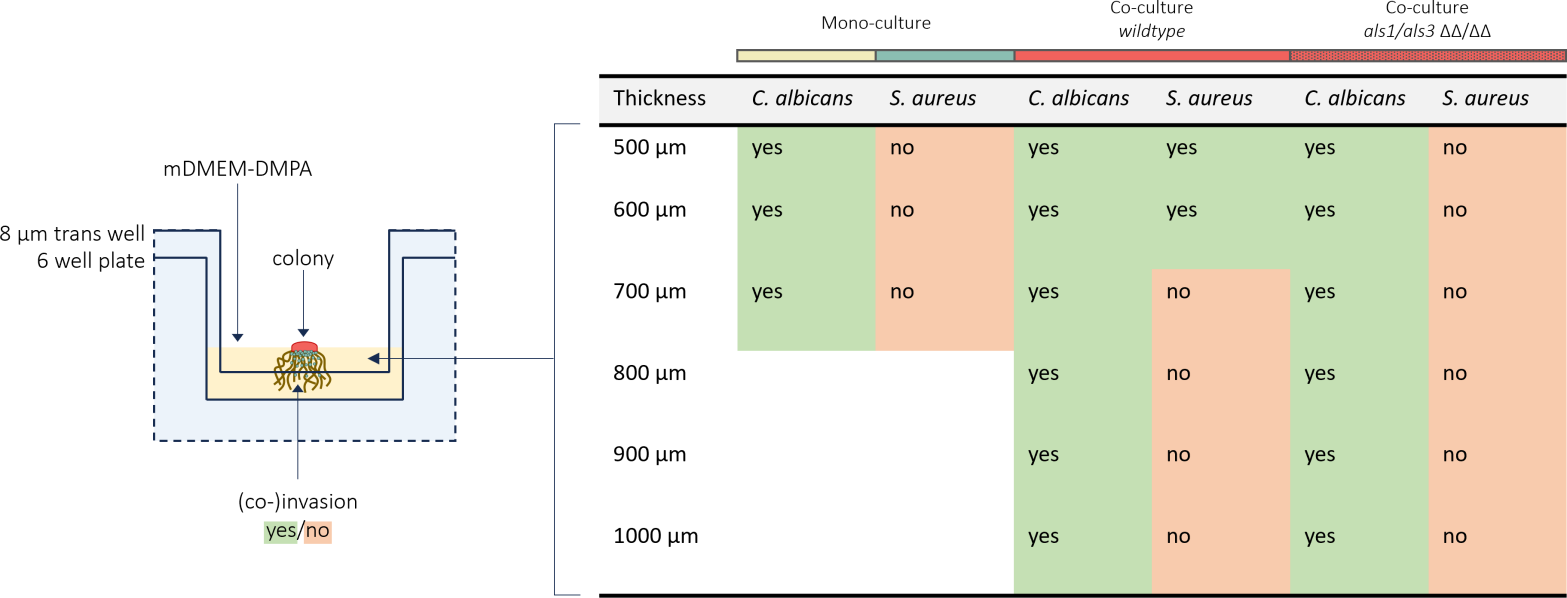
A graphical representation of the *trans*-well-based co-invasion assay setup (left) together with a table showing the results of *in vitro S. aureus–C. albicans* co-invasion over agar layers of various thicknesses in a *trans*-well agar invasion model. Co-invasion was measured for *C. albicans* SC5314 wild-type (yellow) and *S. aureus* ATCC12600 wild-type (blue) monocultures and co-cultures containing either *C. albicans* SC5314 wild-type (red) or als1/als3 ΔΔ/ΔΔ (red, dotted). Successful invasion of each species was measured by observing the presence or absence of growth of the invaded cells on selective plates, which is depicted by either “yes” or “no” within the table. All conditions were tested in biological and technical triplicate and showed unanimous results.

*S. aureus* co-invasion crucially contributes to lethality during the co-infection process and heavily relies on Als1p/Als3p (15–18). However, to study such invasion processes *in vitro,* a semi-solid growth-based co-invasion assay is required, which, to the best of our knowledge, has never been constructed. Therefore, we devised an mDMEM-DMPA *trans* well-based assay in combination with selective plating to study co-invasion. Invasion of wild-type *C. albicans* and *S. aureus* mono- and co-cultures across various thicknesses of mDMEM-DMPA (500, 600, 700, 800, 900, or 1,000 µm) was tested first. To assess whether this construct resembles the *in vivo* dependence on Als1p/Als3p for *S. aureus* co-invasion, mono- and co-cultures of *S. aureus* with *C. albicans* als1/als3 ΔΔ/ΔΔ mutants were tested as well (Fig. 5). Comparable to the *in vivo* situation, monocultured *S. aureus* was not able to invade the underlying substratum.

However, during co-culturing with wild-type *C. albicans*, *S. aureus* was able to co-invade over a distance of 600 µm. Similar to the *in vivo* situation, this co-invasion was no longer observed when *S. aureus* was co-cultured with an als1/als3 ΔΔ/ΔΔ mutant. No differences in invasion were observed between monocultures of wild-type and als1/als3 ΔΔ/ΔΔ *C. albicans* (Fig. 5).

Together, these results show that the constructed *in vitro* mDMEM-DMPA *trans*-well-based co-invasion assay allows for the study of *C. albicans–S. aureus* co-invasion. Moreover, it confirmed that *S. aureus* co-invades during *in vitro* semi-solid growth in an Als1p- and Als3p-dependent manner.

## DISCUSSION

In this study, we investigated the growth and invasion characteristics of *C. albicans* and *S. aureus* mono- and co-cultures on and into semi-solid mDMEM-DMPA. Together, our results uncovered a reciprocal stimulation of growth and (co-)invasion. *C. albicans* was found to promote *S. aureus* growth by maintaining a neutral environmental pH. This promotion was found to occur independent of the pH inducible *S. aureus* agr system. Nevertheless, our recent study has shown that the agr system is still, partly, activated by the pH control of *C. albicans* (41). Aside from promoting *S. aureus* colony expansion, co-culturing with *C. albicans* protected *S. aureus* from shear stress during washing in a hypha-dependent but Als1p/Als3p-independent manner. Conversely, *S. aureus* was found to stimulate *C. albicans* hypha extension and extension rate in a *C. albicans:S. aureus* in a ratio-dependent manner, with a ratio of 1:10 performing best. *S. aureus*-secreted factors accounted for this increase in *C. albicans* hypha formation/invasion. Finally, using a novel *trans*-well-based *in vitro* co-invasion assay, the invading *C. albicans* hyphae were confirmed to facilitate *S. aureus* co-invasion in an Als1p/Als3p-dependent manner, as has been observed *in vivo* in previous studies (16, 17).

Previously, we have shown that *C. albicans* pH maintenance promotes planktonic growth of *S. aureus* (31). Current results show that these findings also hold true for growth on a semi-solid surface. Moreover, although previous research has shown that *C. albicans* is protected against shear stress when co-cultured with *S. aureus* on a solid surface (28), our results show that, when grown on a semi-solid surface, *C. albicans* actually protects *S. aureus* against shear (washing) stress. When hypha growth is inhibited, through Efg1 and Cph1 deletion, this protection is omitted, indicating that *C. albicans* hyphae facilitate this protection. Additionally, hypha growth inhibition leads to an altered appearance of *S. aureus* colony integration, resembling a sunflower-like structure. The formation of this structure is likely attributable to the lack of binding between *C. albicans* and *S. aureus*, possibly creating streams of *S. aureus* flowing down from the rising colony center. Recently, researchers have shown that colony morphology indeed differs between yeast and hyphal *C. albicans:S. aureus* co-cultures and point to *C. albicans* as the main determinant for colony architecture (42). Our results highlight the importance of *C. albicans* morphology in determining colony macrostructure. Nevertheless, the exact impact of this colony structure on virulence remains unknown. While hypha-dependent, the protection of *S. aureus* by the co-culture colony is independent of Als1p and Als3p, suggesting that the colony structure itself provides the shear-stress protection. Altogether, the co-occurrence of *S. aureus* and *C. albicans* inside the oral cavity could not only promote *S. aureus* growth but also protect *S. aureus* from mechanical removal due to, in this case, salivary flows, oral epithelial shedding, and removal during oral hygiene routines (43–45).

When grown on a solid surface, *S. aureus* conversely promoted *C. albicans* hypha growth in a concentration-dependent manner with an optimal *C. albicans:S. aureus* ratio of 1:10. The fact that *C. albicans* hyphae grew significantly shorter during co-cultures with a ratio of 1:100 compared to co-cultures with a ratio of 1:10 is likely attributable to early (0–7.5 h) environmental acidification, which is a known inhibitor of hypha formation (46). Our previous study has shown that *S. aureus* is able to quickly acidify its environment, which is counteracted by the alkalinizing ability of *C. albicans* during co-culturing (31). The 10-fold higher amount of *S. aureus* used here could allow the bacteria to overrule the environmental pH to a more acidic level, which *C. albicans* can overcome by eventually taking back control of the environmental pH, as shown by its increase in average hyphal growth rate to similar levels of the 1:10 co-culture for the final 7.5 h. Ultimately, semi-solid surface experiments showed that the increased *C. albicans* hypha extension was attributable to *S. aureus* secreted factors (≥ 3 kDa). In a previous study, we were able to elucidate the composition of this fraction (41). There, we showed that *S. aureus* indeed promotes the presence of *C. albicans* hyphal-related proteins in the secretome. Furthermore, we confirmed that *C. albicans* significantly increases the presence of *S. aureus* Agr system-related proteins in the secretome, both in a pH-dependent and -independent manner. Nevertheless, the results of our present study suggest that the observed colony size increase during co-culturing is not Agr-dependent and imply that, during unbuffered co-culture growth, the Agr system can reduce growth potency. A previous study has shown that the Agr system produced alpha-hemolysin, which obstructs *C. albicans* yeast-hypha switching, possibly explaining the reduced growth potency (47). However, while they link this obstruction to alpha hemolysin-induced cell cycle arrest (G0/G1 phase) (47), this arrest has also been linked to the induction of hyphal growth (48). Ultimately, while our results do indicate that the Agr system exerts a small inhibitory effect during unbuffered co-culturing, the overall combination of factors still results in a significant increase in hyphal formation and growth. Interestingly, our current results also showed that heat-killed *S. aureus* cells did not affect *C. albicans* hypha expansion, although muramyl dipeptides in bacterial peptidoglycan are known to induce the switch from yeast to hypha growth in *C. albicans* (49, 50). Due to the fact that (i) muramyl dipeptides have a molar mass of 0.492 kDa and should, thus, be filtered out during medium filtration with 3 kDa filter units and (ii) the compound(s) responsible for the hypha induction appeared to diffuse over large surface areas, it is unlikely that the found effect of *S. aureus* on *C. albicans* hypha extension is attributable to muramyl dipeptides. Therefore, while the muramyl dipeptides in the bacterial cell wall promote the switch from yeast to hypha growth, secreted factors could promote the further extension and growth rate of these hyphae.

To expand the functionality of the used semi-solid growth surface, a *trans*-well-based co-invasion assay was constructed and tested. This assay was shown to resemble the *in vivo* situation where *C. albicans* allows for *S. aureus* co-invasion in an Als1p/Als3p-dependent manner (16, 17). The fact that *C. albicans* is able to invade over a larger distance than *S. aureus* shows that hypha invasion precedes the co-invasion of *S. aureus*. Nevertheless, *S. aureus* was able to co-invade over a distance of 600 µm, which, in a human, would be enough to traverse all known oral epithelial layers (51). Epithelial layers are, however, expected to form a stronger barrier compared to the used 0.3% semi-solid agar in our assay, suggesting that the observed invasion will likely be less distant during *in vivo* co-invasion. However, unlike agar, oral epithelial layers are damaged by the lytic enzymes and peptides that are secreted during AP, which would again result in more (co-)invasion (8–11, 17). The mechanisms that drive AP-driven epithelial (co-)invasion, therefore, remain to be elucidated. Our model system provides an easier screening method for *S. aureus* co-invasion, using mutant libraries, compared to complex tissue models. However, agar does not fully resemble the structural complexity, mechanical resistance, or cellular response of host tissues. Future research must show if our constructed semi-solid agar-based model system could be adapted to accommodate research into the contribution of AP on epithelial (co-)invasion of *C. albicans* and *S. aureus*. By replacing the semi-solid mDMEM-DMPA layer present inside the *trans*-well insert with liquid mDMEM-DMP, the system could allow for the cultivation of oral epithelial cells on the top side of the insert membrane and, thus, perform studies with a tri-kingdom setup using this technique. Nevertheless, to our knowledge, this is the first easy-to-use interkingdom co-invasion assay to be constructed and its results shown to resemble *in vivo* findings. Therefore, this model system can be used to study *S. aureus* co-invasion in more detail and test possible methods to prevent staphylococcal co-invasion. Moreover, (co-)invasion of other *Candida* species (e.g., *C. glabrata*) and other bacteria (e.g., *Staphylococcus epidermidis* and *Enterococcus faecalis*) could be investigated as well. The co-invasion of *S. aureus* and potential other bacteria could be the point of initiation for the proposed Trojan horse hypothesis (18, 52), where *C. albicans* facilitates *S. aureus* invasion and intra-tissue phagocytes to take up *S. aureus* and move throughout the lymphatic system, where *S. aureus* survives phagocytic killing, escapes the phagocyte, and disseminates throughout the body.

Altogether, *C. albicans* was shown to create a favorable growth environment for *S. aureus* while protecting growing cells from environmental fluid flows. Meanwhile, *S. aureus* likely promotes its own co-invasion by actively stimulating hypha extension through the production of presently unknown >3 kDa-secreted factors and sequentially using hypha Als1p and Als3p. This illustrates the complexity of interkingdom interactions related to life-threatening BSI.

## SUPPLEMENTAL MATERIAL

Figure S1 aem.01961-25-s0001.tif Colony areas after 72 h of growth concerning *S. aureus* ATCC12600^GFP^, Newman, and 8325-4 monocultures or co-cultures with wild-type *C. albicans* SC5314.

Supplemental legend aem.01961-25-s0002.docx Descriptive legend for Fig. S1.

ASM does not own the copyrights to Supplemental Material that may be linked to, or accessed through, an article. The authors have granted ASM a non-exclusive, world-wide license to publish the Supplemental Material files. Please contact the corresponding author directly for reuse.
