# Supplementary figures and images for: Secreted factors of Staphylococcus aureus promote co-invasion with Candida albicans by inducing hypha formation and invasion
Source: Appl Environ Microbiol. 2026 Mar 31;92(4):e01961-25. doi: 10.1128/aem.01961-25 (PMC13101462; doi:10.1128/aem.01961-25)

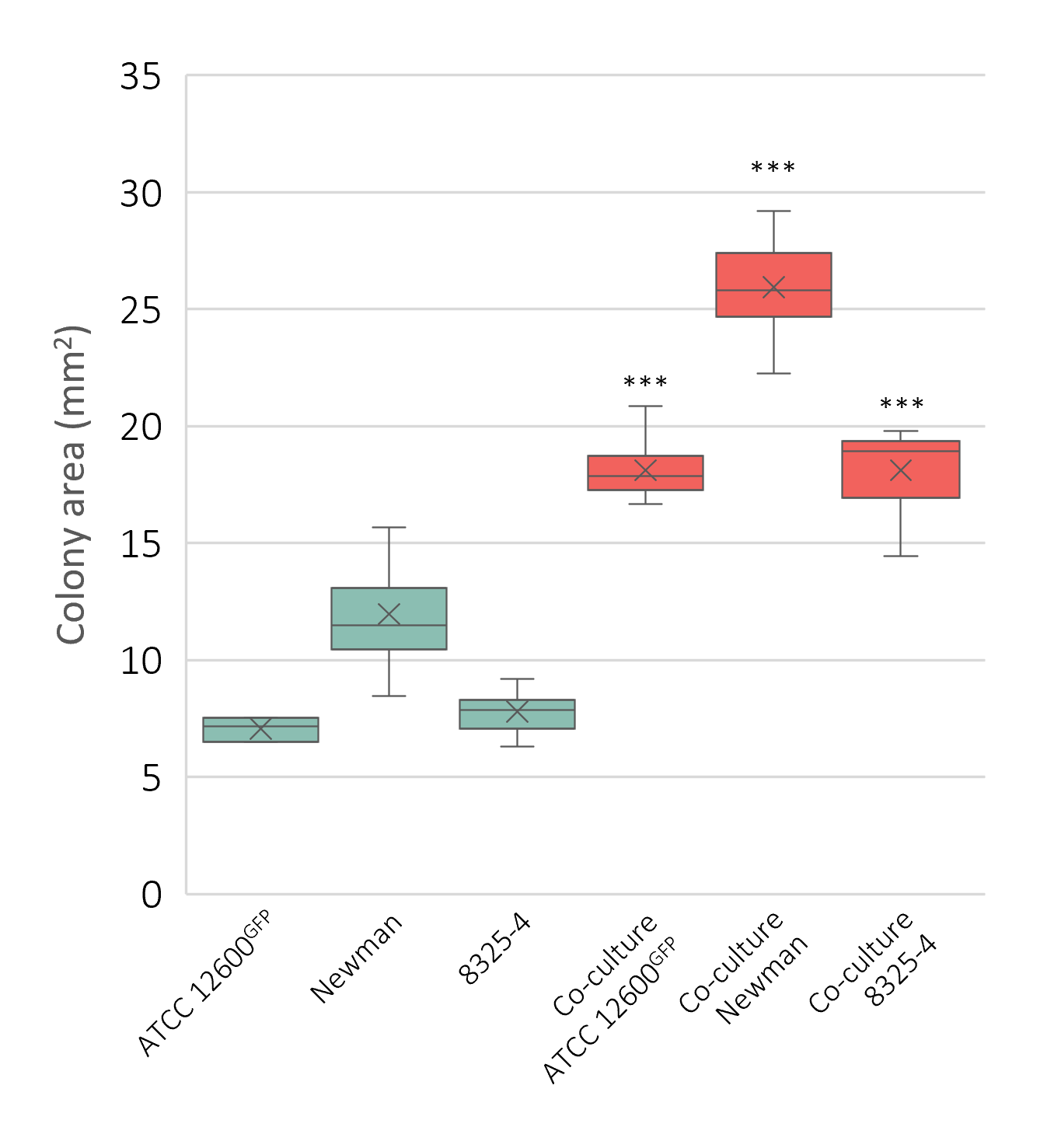

Supplement: Figure S1 — Colony areas after 72 h of growth concerning S. aureus ATCC12600GFP, Newman, and 8325-4 monocultures or co-cultures with wild-type C. albicans SC5314. [file aem.01961-25-s0001.tif]
