# Supplementary material for: Secreted factors of Staphylococcus aureus promote co-invasion with Candida albicans by inducing hypha formation and invasion
Source: Appl Environ Microbiol. 2026 Mar 31;92(4):e01961-25. doi: 10.1128/aem.01961-25 (PMC13101462; doi:10.1128/aem.01961-25)
Supplement: Supplemental legend — Descriptive legend for Fig. S1. [file aem.01961-25-s0002.docx]

Figure S1: Colony areas (mm2) after 72 hours of growth concerning S. aureus ATCC12600^GFP^, Newman, and 8325-4 monocultures (blue) or co-cultures with wildtype C. albicans SC5314 (red). All conditions were tested in biological and technical triplicate with each co-culture condition compared to its corresponding monoculture using ANOVA (***p≤0.001).
